# Overcoming Multidrug Resistance in Cancer Stem Cells

**DOI:** 10.1155/2015/635745

**Published:** 2015-11-16

**Authors:** Karobi Moitra

**Affiliations:** Department of Biology, College of Arts and Sciences, Trinity Washington University, 125 Michigan Avenue NE, Washington, DC 20017, USA

## Abstract

The principle mechanism of protection of stem cells is through the expression of ATP-binding cassette (ABC) transporters. These transporters serve as the guardians of the stem cell population in the body. Unfortunately these very same ABC efflux pumps afford protection to cancer stem cells in tumors, shielding them from the adverse effects of chemotherapy. A number of strategies to circumvent the function of these transporters in cancer stem cells are currently under investigation. These strategies include the development of competitive and allosteric modulators, nanoparticle mediated delivery of inhibitors, targeted transcriptional regulation of ABC transporters, miRNA mediated inhibition, and targeting of signaling pathways that modulate ABC transporters. The role of ABC transporters in cancer stem cells will be explored in this paper and strategies aimed at overcoming drug resistance caused by these particular transporters will also be discussed.

## 1. Introduction

Chemotherapy has long been the method of choice for the treatment of metastatic tumors; however, cancer cells frequently develop an almost uncanny ability to resist the effects of cancer chemotherapeutic agents. This ability of cancer cells to become simultaneously resistant to several structurally unrelated drugs that do not have a common mechanism of action is known as multidrug resistance and can severely impair the success of cancer chemotherapy. Cellular mechanisms of drug resistance arise in the cancer cell itself due to either genetic or epigenetic alterations that can alter sensitivity of the drug. In the clinical setting these may include pumping out of the drug by ABC transporters (ABCB1/P-glycoprotein, ABCC1, ABCG2, etc.), sequestering of drugs into vesicles and subsequent extrusion by exocytosis, and reduced uptake of drugs, such as, water soluble drugs that piggyback on transporters and carriers that are used to bring nutrients into the cell. Other mechanisms are the activation of detoxifying pathways such as the cytochrome P-450 pathway and the cellular glutathione system and mechanisms that repair drug induced damage of cancer cells and disruptions in apoptotic signaling pathways allow cells to become resistant to drug induced apoptotic cell death in cancer cells [1, 2]. In populations of cancer cells exposed to chemotherapy more than one of these mechanisms of multidrug resistance may be present; this phenomenon is known as multifactorial drug resistance.

During treatment, cells susceptible to chemotherapy are killed and generally a few cells in the tumor remain and become resistant to drugs; these resistant cells multiply and the tumor eventually becomes unresponsive to treatment. The unanticipated detection of cancer stem cells in solid tumors has drastically changed our outlook regarding carcinogenesis and chemotherapy. The implication of this discovery is far-reaching and would for all intents and purposes indicate that a self-renewing cancer stem cell population is present in tumors. More importantly these cancer stem cell (CSC) populations unlike other cells are “intrinsically” resistant to chemotherapy. This indicates that surviving cancer stem cells can propagate after chemotherapy and subsequently give rise to tumors [3].

In this review we will focus on the role of ABC (ATP-binding cassette transporters) in cancer stem cell (CSC) drug resistance and discuss strategies for overcoming ABC transporter-mediated drug resistance in CSCs.

## 2. The Cancer Stem Cell Model

The cancer stem cell (CSC) model expounds on the process by which established cancers are able to propagate [4]. The CSC theory proposes that a defined population of cancer cells (known as cancer stem cells) have the “exclusive” property to drive the growth and propagation of a tumor. CSCs can also give rise to progeny that have a limited ability to divide [5]. However, tumorigenic stem cells frequently lack some of the control mechanisms present in normal stem cells where proliferation is tightly regulated and the genomic integrity of the cells is maintained. If the doctrine that tumors contain stem cells is assumed to be correct then we could possibly interpret the accumulation of mutations in these stem cells as the basic “multistep” process of carcinogenesis. The uncanny ability of cancer stem cells to resist chemo- and radiotherapy would lead us to ask the question: Does the “innate” or “intrinsic” resistance of stem cells to radiation and toxins contribute to the failure of some cancer therapies? And ultimately ask: How can we exploit our knowledge of stem cell biology to specifically target CSC's in order to overcome drug resistance and improve therapeutic outcome in the clinic?

## 3. ABC Transporters and Stem Cells

**Figure 1:**
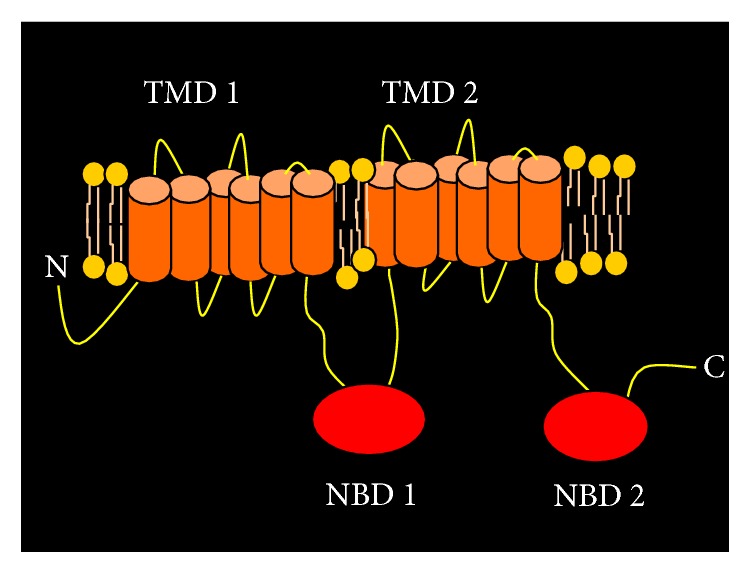
Schematic diagram of a typical ABC transporter (ABCB1/P-glycoprotein) depicting the structural organization of a full transporter. A full transporter typically contains 2 transmembrane domains (TMDs) and 2 nucleotide binding domains (NBDs).

ATP-binding cassette transporters (ABC transporters) are intricate molecular pumps most of which catalyze the transport of a wide array of substrates across biological membranes against a concentration gradient, by the hydrolysis of ATP. The human genome is known to encode 48 ABC transporter genes categorized into seven subfamilies, ranging from A to G [6]. ABC transporters are classified based on the sequence and organization of their ATP-binding domain(s) that contain specific conserved motifs, Walker A and Walker B (present in all ATP-binding protein); however ABC transporters contain an additional motif, the signature motif or the C-loop that is located upstream of Walker B motif. Functional transporters usually contain two transmembrane domains (TMDs) generally made up of 6–12 membrane-spanning alpha-helices that are primarily responsible for determining substrate specificity. Additionally there are two nucleotide binding domains (NBDs) that bind and hydrolyze ATP providing the energy for substrate translocation (Figure 1). ABC transporters may be expressed in stem/progenitor cells derived from several types of normal tissue and also in hematopoietic cells. Hematopoietic stem cells (HSCs) were found to express high levels of ABCG2 and/or ABCB1 transporters [7]. Mouse knockouts for ABCB1, ABCG2, or ABCC1 revealed that mice are particularly sensitive to some compounds such as mitoxantrone, vinblastine, ivermectin, and topotecan indicating that these transporters may have a role in protecting the stem cells from toxic substances [8]. Using the aid of Taqman low density arrays ABC transporters were also found to be expressed in normal stem cells such as hematopoietic stem cells (HSCs), unrestricted somatic stem cells (USSCs), mesenchymal stem cells (MSCs), and multipotent adult progenitor cells (MAPCs) [9]. HSCs seemed to rely on a different repertoire of these transporters compared to other tissue/cell types based on the fact that gene signatures for ABC transporters were found to be radically different between HSCs and other types of stem cells. 16 transporters in total including ABCB1 and ABCG1 were discovered to be consistently expressed at higher levels in HSCs when compared to other transporters. The transporters ABCA4, ABCA8, ABCC9, and ABCG4 were consistently detected in MSCs and USSCs [9].

## 4. Distinguishing Characteristics and Biomarkers of CSCs

Cancer stem cells (CSCs) possess the unique property to evade radiotherapy and chemotherapy. Compared to differentiated tumor cells CSCs have 4 distinguishing features; they are as follows: (i) they are relatively quiescent, (ii) they have a slow cycling rate (the relative quiescence and slow cycling rate afford these cells protection against chemotherapeutics that target rapidly dividing cells rather than a slow cycling subset) [10], (iii) they retain the ability to form tumors when they are injected into nonobese diabetic/severe combined immunodeficiency (NOD-SCID) mice, and (iv) accumulating scientific evidence indicates that cancer stem cells express ABC transporters which confer chemoresistance on this subset of cells [11].

Characteristic cell surface markers and the property of CSCs to exclude certain fluorescent dyes can act as identifying features for stem cells. Cancer stem cells are detected by a number of cell surface markers including CD133 (prominin 1) and CD166 both of which were already known to define stem and progenitor cells. NESTIN (along with CD133) has also been regarded as a CSC marker in disseminated tumor cells of metastatic melanoma patients. In C6 glioma cells it was demonstrated that a small fraction of cells that could form tumor spheres also expressed potential stem cell markers CD133 and NESTIN. In conclusion it can be stated that CD133 is an indicator but may not be a reliable marker for defining CSCs in solid tumors since it does not characterize tumor-initiating cells exclusively and that CD133 is a necessary albeit insufficient criterion to identify CSCs in solid tumors [12]. ABC transporters such as ABCB1, ABCG2, and ABCB5 can also serve as markers of CSCs. ABCB5 expression in tumor cells was seen to correlate with clinical melanoma progression and a subpopulation of human melanoma cells was observed to coexpress ABCB1, ABCB5, and ABCC2 in addition to stem cell markers [12]. Additionally it was found that tumor-initiating cells in human melanoma could be identified by the expression of ABCG2 coexpressed with CD133 [12]. However, it must be kept in mind that tumor-initiating stem cells are for the most part heterogeneous and a specific marker/set of markers has not been found to identify CSCs in solid tumors [12].

## 5. ABC Transporters Define the Side Population in Stem Cells 

The side population (SP) of CSCs is a subset of stem cells that have a high capability for effluxing antimitotic drugs. Cells making up the side population can be isolated by their capacity to efflux fluorescent dye Hoechst 33342 or rhodamine 123 with the help of a flow cytometer. This particular population is called the “side population” because during flow-cytometry analysis these cells can be visualized as a negatively stained population off to “the side” of the main population of cells [13]. The current understanding is that the drug transporting capability of these cells is likely conferred by certain ABC transporters including ABCB1 (rhodamine 123) and ABCG2 (Hoechst 33342). Side population (SP) isolation from a number of cancer cell lines has shown that the expression of* ABCB1* is upregulated in the SP compared to the normal population. SP cells isolated from the PANC1, pancreatic cancer cell line, have been found to express both* ABCB1* and* ABCG2* [14]. The isolated SP cells constituted around 2.1–8.7% of the total population of viable cells identified by Hoechst 33342 staining. This population was found to have an enhanced capacity for the efflux of Hoechst dye that was postulated to be due to ABCB1 and ABCG2 expression. Targeting this side population thus may provide an alternate approach to cancer therapy.

## 6. ABC Transporters as a Causal Determinant of Drug Resistance in Cancer Stem Cells

Clinical drug resistance as we now understand is multifactorial involving alteration in drug targets, inactivation/detoxification of the drug, decreased drug uptake, increased drug efflux, and the dysregulation of apoptotic pathways [1]. ABC transporters including ABCG2, ABCB1, and ABCC1, to name but a few, are known to be associated with drug resistance [3]. ABCG2 is a half transporter postulated to function as a homodimer. It has a molecular weight of 72 kD and a particularly broad substrate-specificity. It has the capacity to transport doxorubicin, mitoxantrone, topotecan, methotrexate, and tyrosine kinase inhibitors among others. Another important transporter is ABCB1 (also known as P-glycoprotein). Its expression has been found in over 50% of all drug resistant tumors. Human ABCB1 is the product of the* MDR1* gene and acts as an ATP dependent pump for a variety of hydrophobic compounds including anticancer and antimicrobial drugs. Side population isolation from cancer cell lines has demonstrated that the expression of* ABCB1* is upregulated in the SP when compared to the normal population. SP cells isolated from the PANC1, pancreatic cancer cell line, have been found to express both* ABCB1* and* ABCG2* [3]. The isolated side population cells were around 2.1–8.7% of the total population of viable cells identified by Hoechst 33342 staining. This side population had an enhanced capacity for the efflux of Hoechst dye which was postulated to be due to ABCB1 and ABCG2 expression [3]. However, apart from ABC transporters there are other factors that may determine drug resistance in cancer stem cells such as the capacity of a stem cell for DNA repair and its quiescent state. Usually tumors which recur after an initial response to chemotherapeutic drugs become resistant to multiple drugs. The cancer stem cell model of drug resistance postulates that the original tumor would contain a small population of tumor stem cells and their differentiated progeny. After exposure to the drug only tumor stem cells (expressing drug transporters) would survive. These stem cells could then divide and repopulate the tumor with stem cells and differentiated cells [13].

## 7. Strategies for Targeting ABC Transporters in Cancer Stem Cells

Cancer stem cells as we know retain the “exclusive” property to drive the growth and spread of a tumor. These cancer stem cells express a number of ABC transporters including ABCG2, ABCB1, ABCB5, and ABCC1. An important strategy to overcome drug resistance in CSCs would be to target the functioning of ABC transporters in these cells. We will discuss the current strategies for inhibiting the function of these transporters that can be applied to CSCs. The strategies are as follows:

- Chemotherapy through competitive and allosteric modulators.
- Chemotherapy mediated by nanoparticle targeting.
- Targeting transcriptional regulation of ABC transporters.
- MicroRNA therapeutics.
- Targeting signaling pathways involved in the regulation of ABC transporters.
- Combinational targeting with CSC targeting agents and transporter modulating drugs or dual targeting with a single agent.

*(i) Chemotherapy through Competitive and Allosteric Modulators.* A directed effort has been devoted to the development of inhibitors against ABC efflux pumps. These inhibitors can be classified as competitive inhibitors that bind to the substrate site and actively compete with the substrate and allosteric modulators that bind at a region that is not the substrate-binding site but can cause a conformational change in the transporter that affects the functioning of the transporter. Historically we can trace the journey of competitive inhibitors from development to clinical application. First-generation inhibitors include drugs like verapamil and cyclosporine that could inhibit the ABCB1 efflux pump. However, the low efficacy of these drugs in the clinical setting necessitated the development of rationally designed inhibitors based on a quantitative structural activity relationship (QSAR). These were termed second-generation compounds and included PSC833 and biricodar (VX-710). However, it was discovered that PSC833 can significantly reduce the clearance of chemotherapeutics and as a result could elevate toxicity of the drug [15]. Thus the clinical trials with these second-generation inhibitors were largely negative probably due to pharmacokinetic interactions between the chemotherapeutics and the P-gp inhibitor. Development of third-generation inhibitors was thus necessitated that could possibly overcome the drawbacks of the previous generation. XR9576 (tariquidar) and LY335979 (zosuquidar) were two such drugs that were developed. XR9576 inhibit both ABCB1 and ABCG2, while LY335979 is a specific inhibitor of ABCB1/P-glycoprotein. Another potential inhibitor is Fumitremorgin C (FTC) that proved to be a very potent inhibitor of ABCG2. However, a major drawback of this particular drug was undesirable neurotoxic effects. Natural products have also been shown to have inhibitory effects on ABC transporters including ABCB1, ABCC1, and ABCG2. These include the polyphenols and curcumin that appear to modulate the effects of ABCB1, ABCC1, and ABCG2 [15]. Another promising modulator is NSC 73306, a thiosemicarbazone derivative. It has been shown to be selectively cytotoxic to cells that overexpress ABCB1/P-glycoprotein [16]. It was also discovered that NSC73306 could inhibit ABCG2 mediated drug resistance to both mitoxantrone and topotecan. It was subsequently postulated that NSC73306 may have a dual mode of action by eliminating both P-gp expressing cells while also being a potent modulator that could resensitize ABCG2 overexpressing cells to chemotherapeutics [17]. Traditionally competitive inhibitors are used to overcome drug resistance; however, it was shown that the efflux of mitoxantrone from the leukemic stem cells could not be efficiently inhibited by the ABCB1 inhibitors verapamil and PSC-833. The researchers hypothesized that differences between leukemic and normal stem cells may have been caused by additional transport mechanisms in the leukemic stem cells [18]. Another approach to the problem of overcoming drug resistance in CSCs would be to utilize allosteric modulators that would not compete with the substrate but would potentially cause a conformational change in the transporter that would affect its function. It has recently been shown that allosteric modulators of transporters may have the potential to inhibit their functioning [19–23]. A group of compounds known as flupentixols that include cis(Z) flupentixol, and trans(E) flupentixol have been found to allosterically inhibit the transporter ABCB1 [19–23]. Studies have indicated that there is evidence of two modulator-specific sites at the lipid protein interface of ABCB1 (P-gp) that demonstrate negative synergy in influencing ATP hydrolysis [19]. These inhibitors/modulators have the potential to target ABC transporters in cancer stem cells. 

*(ii) Chemotherapy Mediated by Nanoparticle Targeting.* Conventional chemotherapy has several drawbacks among which is the fact that the drug cannot be specifically targeted to the tumor and also the problem of drug efflux by ABC transporters. These issues can partially be addressed with the use of nanoparticles. Nanoparticles (NPs) are widely used in drug delivery systems. One or more cytotoxic drugs may be encapsulated within or bound to a nanosphere or nanocapsule that generally has a diameter between 1 and 1000 nm [24]. These nanospheres are usually composed of a semisynthetic biodegradable polymer. PLGA (polylactide-co-glycolide) is one polymer that has been approved by the FDA. Other kinds of nanocarriers may be micelles, liposomes, carbon nanotubules, and so forth. Nanodrug delivery systems (NDDS) reach the tumor cell through molecular-targeted delivery to release the cargo to the affected cell or across the cell membrane and ideally should exhibit very low to zero off-target effects [25, 26]. There have been some studies that support the use of nanoparticles to combat drug resistance. It was observed that there was a significant reduction in tumor size and increased animal survival rate in the rat xenograft glioma model with indomethacin loaded nanocapsules [27]. It was also discovered that Paclitaxel loaded PLA-PEG (polyethylene glycol) ligand conjugated nanoparticles enhanced drug accumulation in MCF-7 cell tumor xenograft model [28]. It was also indicated that nanoparticles loaded with dual drugs seem to have a better therapeutic efficacy. Studies have shown the role of curcumin as a drug resistance modulator enhanced the therapeutic potential of nutlin-3a for targeting multidrug resistant tumors [29] suggesting that loading nanoparticles with both curcumin and nutlin-3a may be more effective. It has been proposed that ABCB1/P-glycoprotein functions as a “hydrophobic vacuum cleaner” pumping out drugs from the lipid bilayer itself before the drug can reach the cytoplasm [30]. In this context ABCB1/P-glycoprotein mediated efflux may be circumvented by coadministration of P-gp inhibitors and anticancer drugs packaged in nanoparticles which have the property to evade P-gp recognition at cell membrane. As a result these nanoparticles have the capability to deliver a drug directly into the cytoplasm. Several studies have shown that this strategy may effectively circumvent transporter-mediated drug efflux. Paclitaxel, a P-gp substrate, and verapamil, a P-gp inhibitor, were encapsulated in PLGA nanoparticles and this could circumvent P-gp-mediated drug efflux in MDR tumor cells [31]. The same group also demonstrated that doxorubicin loaded nanoparticles were able to increase the cellular delivery and therapeutic efficacy of P-gp substrates in P-gp overexpressing cells [32].

It was very recently shown that a doxorubicin-encapsulated polymeric nanoparticle surface (decorated with chitosan) could specifically target the CD44^+^ receptors of stem-like cells in 3D mammary spheroids in xenograft tumor models. This particular design of nanoparticles was seen to increase the cytotoxicity of the doxorubicin 6-fold in comparison to the use of free doxorubicin [33]. Judging from the initial success of the nanoparticle mediated delivery system it would be pertinent to assume that this system may in the future be applied to other tumors exhibiting drug resistance in stem cells. 

*(iii) Targeting Transcriptional Regulation of ABC Transporters.* Another approach to combating drug resistance in CSCs would be to target the transcriptional regulation of ABC transporters. Efforts to combat drug resistance caused by ABC efflux transporters have mainly focused on the use of functional modulators rather than on therapeutic targeting of transcription. Developing drug candidates that could potentially inhibit ABC transporters at the transcriptional level would be an effective mechanism of avoiding drug resistance and could also be an excellent approach to target CSCs which overexpress these efflux pumps. It may be possible to prevent transcriptional activation with the aid of prophylactic intervention. Ecteinascidin 743 (ET-743) can target the transcriptional activation of P-gp (ABCB1/MDR1) in the laboratory. This compound is a natural product (tris)tetrahydroisoquinoline related to the saframycin family of compounds isolated from the sea squirt* Ecteinascidia turbinata*. ET743 is capable of interfering with the activation of MDR1 transcription and basic studies have shown that it may affect the MDR1 enhanceosome complex; however its precise mechanism of action has not been elucidated [34]. However, advances in the elucidation of transcriptional regulation of ABC transporters may pave the way toward the development of novel therapeutic agents. Overexpression of MDR1 in drug resistant cells may be a result of gene amplification or transcriptional overexpression. It was discovered that p53 may have a role in the regulation of the MDR1 gene. Wild-type p53 was found to repress transcription of both endogenous MDR1 gene and MDR1 reporter constructs [35] through direct DNA binding at the HT site. Different p53 family members such as p63 and p73 were found to activate MDR1 transcription through an indirect interaction with the APE site (the alternative p63/p73 element) indicating that p53 DNA binding domains can differentially regulate transcription through both DNA binding dependent and independent mechanisms [36]. C-terminal-binding protein 1 (CtBP-1) can also act as an activator of MDR1 gene transcription and could be a new target for inhibiting MDR1-mediated drug resistance [37]. MDR1 gene expression may be activated by different means such as UV irradiation, sodium butyrate, retinoic acid, HDAC inhibitors, and certain chemotherapeutics. Signals from different stimuli may converge on a region of the MDR1 promoter which has been referred to as the “MDR1 enhanceosome” [34]; thus the enhanceosome would make an attractive therapeutic target. The enhanceosome contains binding sites for a variety of transcription factors. These proteins can recruit P/CAF (a histone acetyl transferase) to the MDR1 promoter region. The result of P/CAF recruitment would be acetylation of promoter-proximal histones followed by transcriptional activity. If the complex mechanistic process of transcription regulation can be understood there is potential to develop agents that prevent the transcriptional activity of a variety of drug transporters. There were a large number (232) of potential regulatory transcription factor binding sites discovered through in silico studies in the promoter region of ABCB5 including CREB, PAX6, CEBP, and OCT1 and also ABCC1 [3]. Functional validation of these transcription factors in ABCB5 and other transporters would be an interesting approach to initiate studies to develop potential inhibitory agents.

*(iv) MicroRNA Therapeutics*. MicroRNAs (miRNAs) are classified as small single stranded RNAs that range from 19 to 25 nucleotides in length and are noncoding. They have the capacity to regulate gene expression by usually binding to the 3′UTR. An “indirect” or “potential” model has been proposed that links miRNAs to the regulation of CSCs [38]. This model proposed states that the aberrant expression of microRNA (oncomiRs, oncogenic miRNA or tumor suppressor miRNA) may result in the dysregulation of certain stem cell genes [38]. The impact of this aberrant expression might be to cause increased self-renewal of CSCs and impaired differentiation of a subset of CSCs. The authors propose that this dysregulation could result in carcinogenesis and oncogenesis [38]. A few miRNAs have been found that regulate ABCB1 and ABCG2 and could be important to ABCB1 and ABCG2 expression in CSCs. hsa-miR-451 was found to regulate ABCB1 in MCF7 breast cancer cells [39]. It was also discovered that both miR-451 and miR-27a could regulate ABCB1 expression in multidrug resistant A2780DX5 and KB-V1 cancer cell lines [40]. It was also observed that on treating K562 human chronic myelogenous leukemia cells with increasing concentrations of imatinib an inverse regulation of ABCG2 expression was seen with both miR-328 and miR-212 [41]. In a reporter gene assay, miR-212 was definitely shown to inversely regulate ABCG2 [41] given that ABCG2 is expressed in CSC's; this could be a very important finding for future therapies. From microRNA profiling in drug sensitive and drug resistant MCF-7 breast cancer cells it was discovered that there was a differential expression of miRNA between these cells. Notably hsa-miR-382, hsa-miR-23b, and hsa-miR-885-5p were upregulated and hsa-mir-218, hsa-miR-758, and hsa-miR-548d-5p were downregulated [42]. An additional study documented that in human breast cancer cells resistant to etoposide (MCF-7/VP-16) miR-326 was downregulated in resistant cells. Subsequently transfection of miR-326 into MCF-7/VP-16 cells downregulated ABCC1 expression and increased the sensitivity to etoposide and doxorubicin [43], suggesting that there are possible microRNA targets that can be investigated to circumvent ABC transporter-mediated drug resistance in CSCs. 

*(v) Targeting Signaling Pathways Involved in the Regulation of ABC Transporters*. Regulating the protein expression of ABC transporters in CSCs may provide an alternative strategy to target ABC transporter functioning in CSCs. The Hedgehog (Hh) pathway is involved in several developmental processes of cells such as the determination of cell fate, patterning, proliferation, survival, and differentiation. In mammals, three Hh proteins (Sonic, Indian, and Desert) are present. Hh acts by binding to Patched (PTCH) [44]. In the absence of ligand, PTCH constitutively represses the activity of a protein called Smoothened (SMO). When Hh ligand binds to PTCH, the repression of SMO is released and the expression and/or posttranslational processing of the three GLI zinc-finger transcription factors is achieved. The Gli proteins are capable of inducing the expression of several target genes [45]. It has been indicated that Hedgehog signaling can regulate the expression of MDR1 and ABCG2 [46]. Treatment of PC3 cells with cyclopamine, a SMO signaling element inhibitor, downregulates the expression levels of MDR1 and ABCG2. Targeting of cancer stem cells holds great promise in treating aggressive cancers. Diverse pathways are involved in stem cell differentiation and renewal including oncogenic cascades like EGFR, hedgehog (HH), WNT-*β*-catenin, and a variety of oncogenic signaling elements including NF-KB, AKT, PI3 kinase, Cox 2, and ABC efflux pumps [47, 48]. These factors play a key role in regulating SC self-renewal, survival, differentiation, and drug resistance and may be viable candidates for molecular targeting. It was recently found that abnormal expression of the hedgehog (Hh) signaling pathway transcription factor Gli1 is involved in the regulation of ABC transporters ABCB1 and ABCG2 in ovarian cancer [49]. It was found that the inhibition of Gli1 expression can decrease ABCB1 and ABCG2 gene expression levels and enhance the response of ovarian cancer cells to specific chemotherapeutics [49]. Thus targeting signaling pathways may provide a directed approach to overcoming drug resistance in CSCs.

*(vi) Combinational Targeting with CSC Targeting Agents and Transporter Modulating Drugs or Dual Targeting with a Single Agent*. Another possible avenue to target CSCs may be with a single compound that targets CSCs and also modulates ABC transporters or with a combination of two different types of drug. In the case of two drugs this could lead to a potentiated effect of the combination of agents. A large family of polyphenolic molecules called flavenoids modulate multidrug resistant transporters and also inhibit CSC growth. The anticancer properties of flavenoids are primarily due to their antimitotic activity and also due to inhibition of certain enzymes. Flavonoids may also inhibit the function of ABC transporters such as MDR1/P-gp, MRPs, and ABCG2/BCRP. Flavenoids are ideal for interaction with these pumps because they are hydrophobic molecules and they display low toxicity but show a broad spectrum of biological activities. One such compound is LY294002. It is a PI3K specific inhibitor and is able to block the osteosarcoma CSC cell cycle (G0/G1) through inducing apoptosis by preventing phosphorylation of PKB/Akt* via *PI3K phosphorylation inhibition. This particular compound also inhibits BCRP, ABCB1/P-gp, and MRP1 [44].

## 8. Conclusions

It is imperative to gain a better understanding of the mechanisms involved in the resistance of stem cells to chemotherapy. Once the mechanism has been understood it can lead to the discovery of new therapeutic targets and improvement of current anticancer strategies. One factor that is responsible for chemoresistance in CSCs is ABC transporters. Based on numerous studies it is apparent that targeting ABC transporters in CSCs can lead to a better outcome for patients given that according to the cancer stem cell hypothesis these stem cells are the only cells in the tumor capable of giving rise to a new tumor. The stem cell model of drug resistance is an important step forward in the field of cancer drug resistance because it gives us an avenue to explain how cancers which show an apparent complete clinical response to chemotherapy can relapse either within months or even years later. In order to elucidate and improve upon this model, however, it is necessary to define stem cells by their long-term self-renewal potential and not just by the existence of a side population. The simple fact that we can now identify, purify, and propagate cancer stem cells may in the future allow us to develop new strategies to improve targeted cancer therapeutics and thereby improve patient outcomes.
